# Editorial: Targeting Excitation-Inhibition Imbalance in Neurodevelopmental and Autism Spectrum Disorders

**DOI:** 10.3389/fnins.2022.968115

**Published:** 2022-07-08

**Authors:** Susanna Pietropaolo, Giovanni Provenzano

**Affiliations:** ^1^INCIA, UMR5287, Bordeaux University and CNRS, Bordeaux, France; ^2^Department of Cellular, Computational and Integrative Biology – CIBIO, University of Trento, Trento, Italy

**Keywords:** developmental disabilities, excitatory/inhibitory balance, animal models, pharmacologic (drug) therapy, Autism Spectrum Disorders (ASDs)

A tight balance between excitation and inhibition (E/I balance) in synaptic inputs to a neuron and in neural circuits is important for correct brain development and function. E/I imbalances have been implicated in numerous brain and neurodevelopmental disorders, such as Autism Spectrum Disorders (ASDs). ASDs refer to a wide range of neurodevelopmental disorders and a variety, a “spectrum” of symptoms, skills, and levels of disability. Although most ASDs present without an apparent cause (i.e., idiopathic), “syndromic forms” are frequent and result from single-gene defects. They include disorders such as Tuberous Sclerosis Complex 1 and 2 (TSC 1 and 2), Fragile X syndrome (FXS), Rett syndrome (RTT). Several studies, both at cellular and circuit level, have hypothesized that some of these syndromic forms of ASD might be caused by an excitation/inhibition (E/I) imbalance.

Neuronal E/I balance is established and tightly regulated by several factors at synaptic or circuit levels. Specific factors that contribute to synaptic E/I balance include excitatory/inhibitory synapse development, synaptic transmission and plasticity, downstream signaling pathways, homeostatic synaptic plasticity, and intrinsic neuronal excitability. At the circuit level, E/I balance involves local circuits such as the interplay between GABAergic interneurons and target pyramidal neurons, which modulate long-range connections. Although several studies point to a local hyperconnectivity and long-range hypoconnectivity and disconnection between neural circuits in ASD, the pathogenic mechanisms underlying E/I imbalance in ASDs might be more complex than expected. Understanding the different cellular environments and finely regulated time-windows influencing E/I imbalance in ASDs brains is not only required to improve basic understanding of the causal link between E/I imbalance and the initiation, development and maintenance of autistic-like phenotypes, but has also important implications for developing better pharmacological treatments.

Pharmacological strategies for ASD targeting E/I imbalance are reviewed in the article by Canitano and Palumbi summarizing clinical studies on pharmacological agents that are used to restore the cortical balance of excitation and inhibition in ASD patients. Among these compounds the authors focused on glutamatergic and GABAergic drugs, e.g., acting on NMDA receptors (e.g., the NMDA antagonist memantine), or GABA receptors (e.g., STX209 and bumetanide) showing promising efficacy to treat selective ASD symptoms, namely irritability, and some social and communication abnormalities. Beside these “classical” molecules, novel therapeutic approaches based on modulators of E/I imbalance are proposed in the review, including a modified form of memantine, i.e., nitrosynapsin, and oxytocin, because of its role in GABA-mediated E/I control of social behaviors, but also non-pharmacological strategies, such as transcranial magnetic stimulation (TMS).

The therapeutic efficacy of acting on GABA receptor functionality has been further addressed in the study by Juarez-Martinez et al., investigating the effects of Bumetanide in patients affected by Tuberous sclerosis complex (TSC). TSC is a monogenetic neurodevelopmental disorder due to a loss-of-function mutation in the mTOR pathway regulators TSC1 or TSC2 (tumor suppressor) genes leading to intellectual disability, ASD-related behaviors and epilepsy. Since neuronal E/I imbalances in terms of a reduced inhibition have been implicated in TSC, therapies based on Bumetanide have been suggested, since this agent may induce a downregulation of intraneuronal chloride concentration, with a shift in GABA polarity from depolarizing (with excitatory activity) to hyperpolarizing (inhibitory activity). This study assessed resting-state EEGs and behavioral alterations in children with TSC before and after 3 months of bumetanide treatment. At baseline, TSC patients showed lower alpha-band absolute power and functional E/I ratio (E/I) compared to typically developing children and this deficit was reduced after bumetanide treatment, together with a moderate, behavioral improvement. Interestingly, the most marked responsiveness to the treatment was observed in children with network characteristics around the E/I balance point, thus suggesting that baseline network characteristics might influence treatment efficacy and highlight the utility of E/I-sensitive EEG measures to test new therapeutic interventions for TSC.

The GABAergic system has been the target also of the study by Budimirovic et al. assessing the effects of a GABA-agonist, Gaboxadol (at multiple doses), in patients with another monogenic syndrome linked to ASD, the Fragile X syndrome (FXS). FXS is an inherited neurodevelopmental disorder caused by a full-mutation expansion in the promoter region of the fragile X mental retardation 1 (FMR1) gene, resulting in the absence of fragile X mental retardation protein (FMRP) (Bagni et al., 2012). FXS is also the most common single-gene cause of intellectual disability and ASD. Although these preliminary results need to be confirmed in a larger, randomized, placebo-controlled study, overall, the authors demonstrated that Gaboxadol administered once a day was safe and well tolerated and had an encouraging behavioral efficacy.

The therapeutic value of targeting the GABAergic system has been further evaluated in the article by Li, focusing on another neurodevelopmental disease associated with ASD, i.e., Rett syndrome (RTT). This article provides a review of pre-clinical and clinical evidence supporting the efficacy of pharmacological modulation of GABAergic activity in recovering cellular, network, and behavioral dysfunction in RTT mouse models and patients. RTT is the leading cause of intellectual disabilities in girls, and it is caused by loss-of-function mutations in the X-linked gene encoding a transcriptional regulator known as methyl-CpG-binding protein 2 (MeCP2). Individuals with RTT are characterized by a multitude of neurological symptoms, including autistic behaviors, breathing irregularities, and seizures. Beside GABAergic drugs, treatments that have been proposed for RTT include glutamatergic agents, Brain-Derived Neurotrophic factor (BDNF) mimetics, anti-depressant and mitochondria-targeting compounds: their efficacy in cellular and animal models of RTT is extensively discussed by Li, highlighting the relevance of the effects of these molecules on E/I imbalance.

Despite the translational issues concerning animal models of ASD, e.g., high phenotypic variability, lack of a biomarker, and limitations in assessing social complex behaviors, this special issue underlines the bridge between preclinical studies and clinical testing. Pre-clinical models have provided a major contribution in uncovering the neural and molecular bases of E/I imbalance in several neurodevelopmental syndromes associated with ASD, and paved the way to clinical trials that start identifying novel pharmacological treatments, as demonstrated by the clinical studies collected here.

## Author Contributions

All authors listed have made a substantial, direct, and intellectual contribution to the work and approved it for publication.

## Conflict of Interest

The authors declare that the research was conducted in the absence of any commercial or financial relationships that could be construed as a potential conflict of interest.

## Publisher's Note

All claims expressed in this article are solely those of the authors and do not necessarily represent those of their affiliated organizations, or those of the publisher, the editors and the reviewers. Any product that may be evaluated in this article, or claim that may be made by its manufacturer, is not guaranteed or endorsed by the publisher.
